# Syntaxin 1B, but Not Syntaxin 1A, Is Necessary for the Regulation of Synaptic Vesicle Exocytosis and of the Readily Releasable Pool at Central Synapses

**DOI:** 10.1371/journal.pone.0090004

**Published:** 2014-02-28

**Authors:** Tatsuya Mishima, Tomonori Fujiwara, Masumi Sanada, Takefumi Kofuji, Masami Kanai-Azuma, Kimio Akagawa

**Affiliations:** 1 Department of Cell Physiology, Kyorin University School of Medicine, Mitaka, Tokyo, Japan; 2 Radio Isotope Laboratory, Kyorin University School of Medicine, Mitaka, Tokyo, Japan; 3 Department of Anatomy, Kyorin University School of Medicine, Mitaka, Tokyo, Japan; Virginia Tech Carilion Research Institute, United States of America

## Abstract

Two syntaxin 1 (STX1) isoforms, HPC-1/STX1A and STX1B, are coexpressed in neurons and function as neuronal target membrane (t)-SNAREs. However, little is known about their functional differences in synaptic transmission. STX1A null mutant mice develop normally and do not show abnormalities in fast synaptic transmission, but monoaminergic transmissions are impaired. In the present study, we found that STX1B null mutant mice died within 2 weeks of birth. To examine functional differences between STX1A and 1B, we analyzed the presynaptic properties of glutamatergic and GABAergic synapses in STX1B null mutant and STX1A/1B double null mutant mice. We found that the frequency of spontaneous quantal release was lower and the paired-pulse ratio of evoked postsynaptic currents was significantly greater in glutamatergic and GABAergic synapses of STX1B null neurons. Deletion of STX1B also accelerated synaptic vesicle turnover in glutamatergic synapses and decreased the size of the readily releasable pool in glutamatergic and GABAergic synapses. Moreover, STX1A/1B double null neurons showed reduced and asynchronous evoked synaptic vesicle release in glutamatergic and GABAergic synapses. Our results suggest that although STX1A and 1B share a basic function as neuronal t-SNAREs, STX1B but not STX1A is necessary for the regulation of spontaneous and evoked synaptic vesicle exocytosis in fast transmission.

## Introduction

Neurotransmitter release is mediated by the soluble N-ethylmaleimide-sensitive factor attachment protein receptor (SNARE) complex [1], [2], located at the vesicle membrane (v-SNARE) and at the target membrane (t-SNARE) of the presynaptic terminal. The process involves several steps: (1) docking, the initial contact between the synaptic vesicles and plasma membrane; (2) priming, the maturation process that confers responsiveness to the initial influx of Ca^2+^; and (3) fusion of the synaptic vesicles with the plasma membrane [3]. Syntaxin 1 (STX1) forms a ternary SNARE protein complex by interacting with synaptosomal-associated protein of 25 kDa (SNAP-25) and vesicle-associated membrane protein-2 (VAMP-2)/synaptobrevin. STX1 also binds to numerous presynaptic proteins, such as Munc18-1 [4], Ca^2+^-channels [5], [6] and complexin [7]. Two STX1 isoforms are coexpressed in the CNS: HPC-1/STX1A and STX1B [8], [9].

Loss of VAMP-2 or SNAP-25 results in severe impairment of Ca^2+^-evoked synaptic vesicle exocytosis but has a relatively mild effect on spontaneous vesicle release [10], [11]. Previously, we reported that deletion of STX1A has no effect on evoked or spontaneous fast synaptic transmission, although impairments are seen in hippocampal monoaminergic transmission, long-term potentiation and learning ability [12], [13]. This phenotype might be due to compensation by STX1B, because these isoforms are highly homologous. However, the physiological differences between these isoforms in neuronal function remain unknown. In the present study, we examine the function of the STX1 isoforms in fast synaptic vesicle exocytosis by analyzing neural phenotypes of STX1B null neurons and STX1A/1B double null neurons in culture. We show that although STX1A and 1B share a basic function as neuronal t-SNAREs, STX1B is the principal mediator for spontaneous and evoked fast synaptic vesicle exocytosis.

## Materials and Methods

### Ethics statement

The animal experimentation described here was approved by the Animal Care Committee of Kyorin University.

### Generation of syntaxin 1B knockout mice

A BAC clone (37L7) containing a mouse syntaxin 1B gene was purchased from Invitrogen (Carlsbad, CA). A 13-kbp fragment containing exons 4–10 was subcloned into pBlueskript. The region from exon 9 to exon 10 that encoded the H3 and transmembrane domains was replaced with a neomycin-resistant gene, and the diphtheria toxin gene was attached to the 3′ end of the construct. The linearized targeting vector was transfected into ES cells, which were selected from a culture medium containing G418. G418-resistant ES cell colonies were screened by PCR and Southern blot analysis. The knockout ES cell clones were injected into blastocysts and implanted into pseudopregnant ICR females. The resultant chimeric mice were bred with C57Bl/6J to generate heterozygous mutant mice, and genotypes of the offspring were determined by PCR analysis. Finally, the heterozygote was backcrossed with C57Bl/6J for four to five generations [14].

### Cell culture

WT and STX1B^−/−^ neurons from postnatal day 0 hippocampus were dissociated by trypsin (5 mg/ml for 15 min at 37°C), triturated with a siliconized pipette, plated on glial feeder layer at a density of 3–4×10^4^/cm^2^, and cultured in DMEM containing 10% fetal bovine serum at 37°C in a humidified incubator with 95%-air, 5%-CO2. After culture in vitro for 24 h, the medium was replaced with DMEM containing 2% B-27 supplement and 2 µM Ara-C, and used at 14–21 days in vitro. STX1A/STX1B double null neurons from E12.5 cortex were plated on a WT glial feeder layer or glass coverslips coated with polyethyleneimine, using a whole cortex per 35 mm dish. Neurons were used at 21–28 days in vitro.

### Electrophysiology

Synaptic responses were monitored with dual whole-cell patch clamp recordings from pre- and post-synaptic neurons at room temperature as reported [15]. Recordings were made in modified Tyrode solution (mM); 135 NaCl, 3.5 KCl, 2.0 CaCl_2_, 1.0 MgCl_2_, 10 HEPES, 10 Glucose. Internal solution for presynaptic neurons (mM): 140 K-gluconate, 5 KCl, 10 HEPES, 1 EGTA, 0.1 CaCl_2_, 2 MgCl_2_, 2 MgATP, 0.2 NaGTP; internal solution for evoked postsynaptic responses (mM): 110 K-gluconate, 30 KCl, 5 QX-314, 10 HEPES, 1 EGTA, 0.1 CaCl_2_, 2 MgCl_2_, 2 MgATP, 0.2 NaGTP; internal solution for spontaneous miniature postsynaptic responses (mM): 50 K-gluconate, 90 KCl, 5 HEPES, 1 EGTA, 0.1 CaCl_2_, 2 MgCl_2_, 2 MgATP, 0.2 NaGTP. GABA-receptor mediated responses were monitored in the presence of 10 µM CNQX and 50 µM APV, and AMPA-receptor mediated responses were monitored in the presence of 10 µM Bicuculine and 50 µM APV in the medium. Evoked EPSCs were monitored with 10–15 nM tetrodotoxin (TTX) to inhibit spontaneous bursts of synaptic activities. Both presynaptic and postsynaptic neurons were voltage clamped at a holding potential of −70 mV. Monosynaptic excitatory and inhibitory postsynaptic currents were evoked with a short latency (1–3 msec) when the presynaptic neuron was stimulated by stepping to 0 mV for 3 msec. Spontaneous “mini” release was recorded with 1 µM TTX in the medium and analyzed off-line, using an analysis software MiniAnalysis (Synaptosoft, Decatur, GA). For sucrose stimulation, a hypertonic solution prepared by adding 500 mM sucrose to the Ca^2+^-free Tyrode solution was perfused directly into the region containing the patched cell for 15 second. Series resistance was monitored continuously throughout all experiments by measuring the capacitive current response to a 5-mV voltage step, and was compensated 60%. If resistance changed by more than 10%, the experiment was discarded. Signals were analyzed using Igor Pro 6.3 (WaveMetrics, Lake Oswego, OR). All data are presented as the means ±SEM. Statistical significance was determined using one-way ANOVA and two-tailed Student's *t* tests. Regression lines were compared using an analysis of covariance (ANCOVA).

### Electron microscopy

For electron microscopic analysis of the hippocampal synaptic structure, brains obtained from 7-day-old male mice were fixed with 2.5% glutaraldehyde. These were further fixed with OsO4, and uranyl acetate staining was performed as described previously [16]. The quantitative analysis was performed using NIH Image J as described by Leenders et al. (2001) [17]. Asymmetric synapse with clearly visible active zone from WT and STX1B^−/−^ mice were selected randomly for analysis. The distance between the center of each synaptic vesicle and the active zone was measured. Docked vesicles were defined as located within 50 nm distance from the active zone.

## Results

### Generation of syntaxin 1B knockout mice

**Figure 1:**
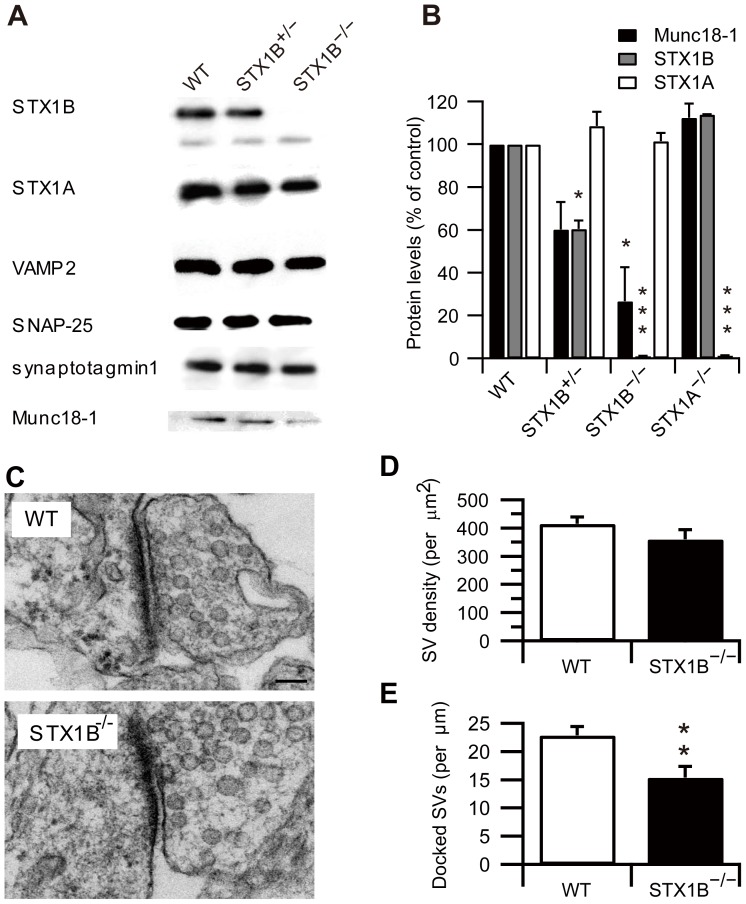
STX1B is required for maintenance of Munc18-1 and vesicle docking. (A) Western blot analysis of STX1A, STX1B and other presynaptic proteins in STX1B^+/−^ and STX1B^−/−^ mice. Whole-brain homogenate was prepared from P7 mice. (B) Expression levels of Munc18-1, STX1A and STX1B from STX1 mutant mice (WT, *n* = 3; STX1B^+/−^, *n* = 5; STX1B^−/−^, *n* = 4; STX1A^−/−^, *n* = 4; one-way ANOVA with Tukey's test). (C) Electron micrographs of the 7-day-old hippocampus of WT and STX1B^−/−^ synapses. Scale bar: 100 nm. (D) Number of synaptic vesicles per µm^2^ of presynaptic terminal in WT and STX1B^−/−^ synapses (WT, *n* = 55; STX1B^−/−^, *n* = 54; *p* = 0.19, two sample *t*-test). (E) The number of docked synaptic vesicles per µm of active zone length. The number of docked vesicles was significantly lower in STX1B^−/−^ synapses than those of WTs (WT, *n* = 15; STX1B^−/−^, *n* = 14; *p* = 0.006, two sample *t*-test). (F) Distribution of SVs. The distribution was significantly different at 0–50 nm from active zone in STX1B^−/−^ synapses (WT, *n* = 15; STX1B^−/−^, *n* = 14; *p* = 0.03, two sample *t*-test). (G) Synapse density in hippocampal CA1 region (WT, *n* = 55; STX1B^−/−^, *n* = 54; *p*<0.001, two sample *t*-test).* *p*<0.05, ** *p*<0.01 and *** *p*<0.001.

Neonatal STX1B null (STX1B^−/−^) mice were smaller than their wild-type (WT) littermates and, unlike STX1A^−/−^ mice, died by postnatal day (P) 14. Western blot analysis of whole-brain extract confirmed that STX1B^−/−^ mice completely lacked STX1B protein, and STX1B^+/−^ mice showed approximately 50% less STX1B protein than WT mice. We examined whether the loss of the STX1B gene would affect the expression of other SNARE-related proteins. The contents of STX1A, SNAP25, VAMP2 and synaptotagmin 1 remained unchanged in STX1B^−/−^mice (Fig. 1A), but Munc18-1 expression decreased with STX1B gene dosage (Fig. 1B).

Electron microscopy was used to study synaptic structure (Fig. 1C). Quantitative analysis of synapses of the stratum radiatum of the hippocampal CA1 region revealed that the total number of synaptic vesicles in presynaptic terminals was not significantly different between WT and STX1B^−/−^ mice (Fig. 1D). However, the number of docked vesicles, defined as located within 50 nm from the active zone (Fig. 1E and F) and synaptic density was significantly lower in STX1B^−/−^ mice (Fig. 1G).

### Syntaxin 1B deletion reduces the frequency of spontaneous neurotransmitter release at glutamatergic and GABAergic synapses

**Figure 2:**
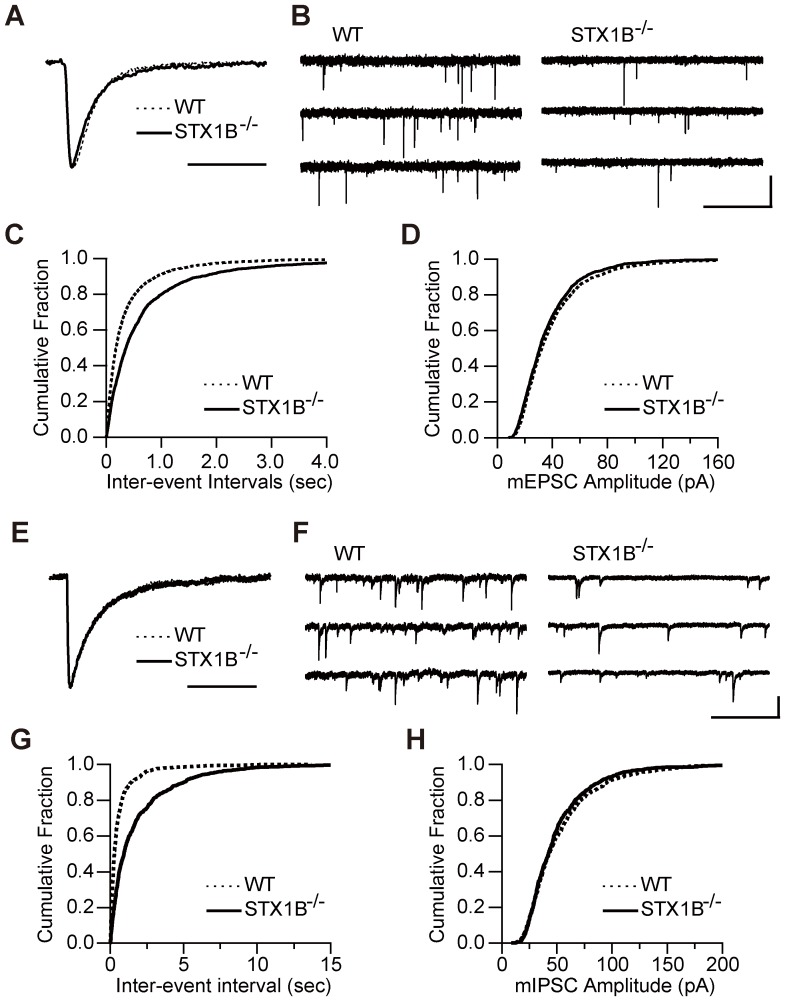
Deletion of STX1B decreases frequency of mEPSCs and mIPSCs. (A) Representative mean mEPSC waveforms in WT and STX1B^−/−^ synapses. Scale bar: 10 ms. (B) Representative mEPSCs. Scale bar: 1 s, 100 pA. (C) Cumulative fraction of the distribution of mEPSC frequency. Frequency of mEPSCs was significantly lower in STX1B^−/−^ neurons (*n* = 33; WT: *n* = 45; *p*<0.001, Kolmogorov-Smirnov test). (D) Cumulative fraction of the distribution of mEPSC amplitude. Mean amplitudes of mEPSCs are 36.9±0.30 pA and 37.9±0.55 pA in WT and STX1B^−/−^, respectively. (E) Representative mean mIPSC waveforms in WT and STX1B^−/−^ synapses. Scale bar: 40 ms. (F) Representative mIPSCs. Scale bar: 1 s, 100 pA. (G) Cumulative fraction of the mIPSC frequency distribution. Frequency of mIPSCs was significantly lower in STX1B^−/−^ neurons (*n* = 52; WT: *n* = 29; *p*<0.001, Kolmogorov-Smirnov test). (H) Cumulative fraction of the mIPSC amplitude distribution. Average amplitudes of mIPSCs: WT, 53.38±0.48 pA; STX1B^−/−^, 50.49±0.44 pA.

Next we investigated the effect of STX1B deletion on spontaneous synaptic postsynaptic currents (PSCs) in culture. We analyzed AMPA receptor-mediated spontaneous miniature excitatory PSCs (mEPSCs) and GABA_A_ receptor-mediated spontaneous miniature inhibitory PSCs (mIPSCs). The kinetics of the mEPSCs and mIPSCs that can be seen from a comparison of the peak-scaled average waveforms of miniature postsynaptic currents (mPSCs) and the mean amplitude of mPSCs did not differ between neurons of WT or STX1B^−/−^ mice (Fig. 2A, D, E and H). However, the mean frequency of mEPSCs (Fig. 2C; WT: 2.68±0.69 Hz; STX1B^−/−^: 1.38±0.19 Hz; *p*<0.001, two sample *t*-test) and mIPSCs (Fig. 2G; WT: 3.45±0.37 Hz; STX1B^−/−^: 0.95±0.14 Hz; *p*<0.001, two sample *t*-test) were significantly lower in STX1B^−/−^ neurons. These results suggest that the deletion of STX1B only affects presynaptic function, and that functional compensation did not occur in the postsynaptic site.

### Syntaxin 1B deletion alters the properties of evoked neurotransmitter release at glutamatergic and GABAergic synapses

**Figure 3:**
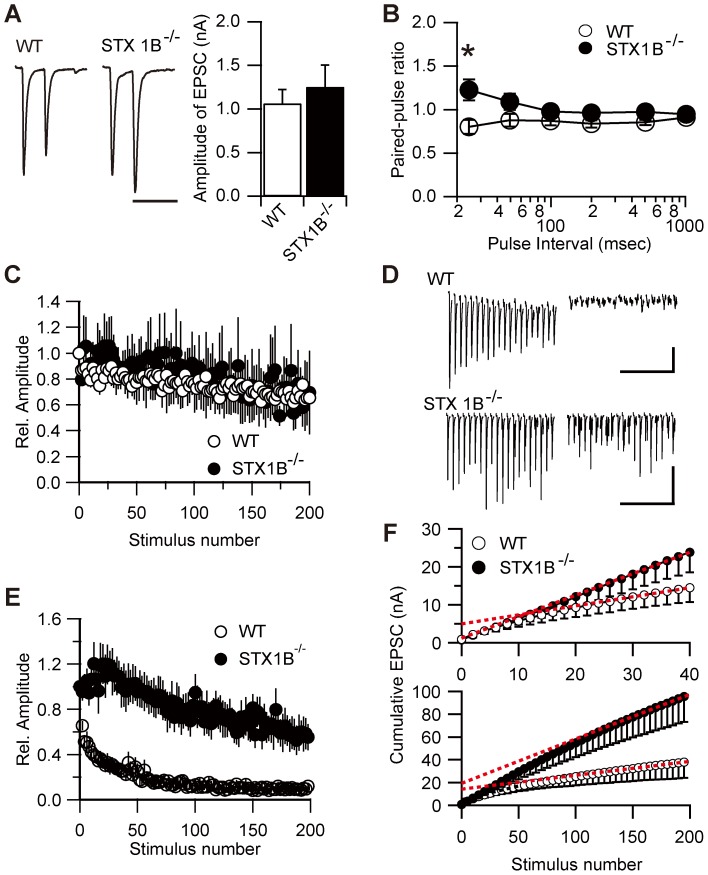
Properties of evoked EPSCs in STX1B^−/−^ neurons. (A) Representative mean eEPSC waveforms in WT and STX1B^−/−^ synapses (left). Neurons were stimulated at 25 ms intervals. Amplitude of AMPA receptor-mediated eEPSCs of STX1B^−/−^ (*n* = 18) and WT (*n* = 22) neurons (right). Scale bar: 50 ms. (B) Paired-pulse ratio of EPSCs was significantly different in cultured neurons from STX1B^−/−^ and WT mice (WT, *n* = 19; STX1B^−/−^, *n* = 14; *p* = 0.049, one-way repeated measure ANOVA with two sample *t*-test). (C) Amplitude of individual eEPSCs during the stimulus train triggered by 200 stimuli at 1 Hz from STX1B^−/−^ (*n* = 7) and WT (*n* = 16) mice, normalized to the amplitude of first response and plotted as a function of stimulus number. (D) Representative eEPSC train stimulated by 20 Hz, recorded from a representative WT (top) and STX1B^−/−^ (bottom) neuron. The first (left) and last (right) twenty responses are presented. Stimulus artifacts were truncated. Scale bar: 0.5 s, 0.5 nA. (E) Amplitude of individual eEPSCs during the stimulus train triggered by 200 stimuli at 20 Hz. A significant difference between neurons from STX1B^−/−^ and WT mice was observed (WT, *n* = 7; STX1B^−/−^, *n* = 8; *p*<0.001, one-way repeated measure ANOVA). (F) Mean cumulative EPSC amplitude value from 40 stimuli, 20 Hz trains in WT and STX1B^−/−^ neurons. Data points between stimuli 30 and 40 (top) and 180 and 200 (bottom) were fitted by linear regression and back-extrapolated to time 0 to estimate the vesicle pool size.

To investigate whether deletion of STX1B would affect evoked neurotransmitter release, we analyzed evoked excitatory PSCs (eEPSCs) using double-patch whole-cell recordings from paired hippocampal neurons in culture. Without the presence of AMPA receptor blockers, the neurons showed high frequency spontaneous epileptiform discharges, and stimulation of the presynaptic neuron caused recurrent EPSCs which interfered with the recordings of eEPSCs. To suppress recurrent eEPSCs, 10–15 nM tetrodotoxin (TTX) was also added to the bath solution; the application of TTX did not affect the properties of transmitter release (data not shown). The mean amplitude of eEPSCs was not different between WT and STX1B^−/−^ mice (Fig. 3A). We also examined short-term plasticity by measuring the paired-pulse ratio at different interstimulus intervals. Loss of STX1B resulted in a higher paired-pulse ratio (Fig. 3B). A reduction in release probability alone does not explain an increase in paired-pulse ratio without a corresponding decrease in amplitude of eEPSCs; therefore, we hypothesized that the effective pool size of excitatory synapses might be greater in STX1B^−/−^ neurons. To examine this possibility, we estimated the effective size of vesicle pool in the readily releasable pool (RRP) by measuring responses to repetitive stimulation at different frequencies. Fig. 3C and E show the time course of synaptic depression by stimulation at 1 and 20 Hz, respectively. There was no difference between WT and STX1B^−/−^ neurons in the profiles of responses to a 1 Hz train of 200 stimuli (Fig. 3C). At 20 Hz stimulation, a rapidly initiating depression occurred over the first few stimuli, followed by a slower depression and a steady-state response in WT (Fig. 3D and E). But neurons from STX1B^−/−^ mice showed initial facilitation until approximately 40 stimuli, followed by overall reduced depression relative to WT neurons during a 20 Hz train. We calculated the cumulative amplitude of EPSCs and determined the effective pool size by fitting a line to stimuli 30–40 and back-extrapolating to the *y*-axis for each group (Fig. 3F top). The *y*-intercepts of the regression lines indicate the estimated effective pool size, and the slopes indicate the vesicle recycling rate [18]. Although the effective pool size of STX1B^−/−^ neurons was difficult to estimate because the responses did not reach an appreciable steady state by the 40th stimulus, the vesicle recycling rate was significantly higher in STX1B^−/−^ neurons (Fig. 3F top; WT, *n* = 7; STX1B^−/−^, *n* = 8; *p*<0.001, two sample *t*-test). The depression phase in Fig. 3E is thought to represent depletion of the vesicle pool, and the steady state represents ongoing replenishment of the depleted pool. It is therefore unlikely that the pool size of excitatory synapses is significantly lower in STX1B^−/−^ neurons, because eEPSC amplitude in STX1B^−/−^ neurons indicated initial facilitation rather than depression. It has been reported that this commonly used method may underestimate the pool size, perhaps because of the presence of “reluctantly releasable” vesicles [19]. To further analyze the pool size, cumulative plots were made from the 200-stimulus trains, with linear extrapolations back to time 0 (Fig. 3F bottom). Estimated pool sizes were not different between WT and STX1B^−/−^ neurons (*p* = 0.41, two sample *t*-test), whereas the vesicle recycling rate was significantly greater in STX1B^−/−^ neurons (*p* = 0.011, two sample *t*-test). These results suggested that the deficiency in STX1B accelerated the replenishment of synaptic vesicles during evoked glutamatergic synaptic transmission.

**Figure 4:**
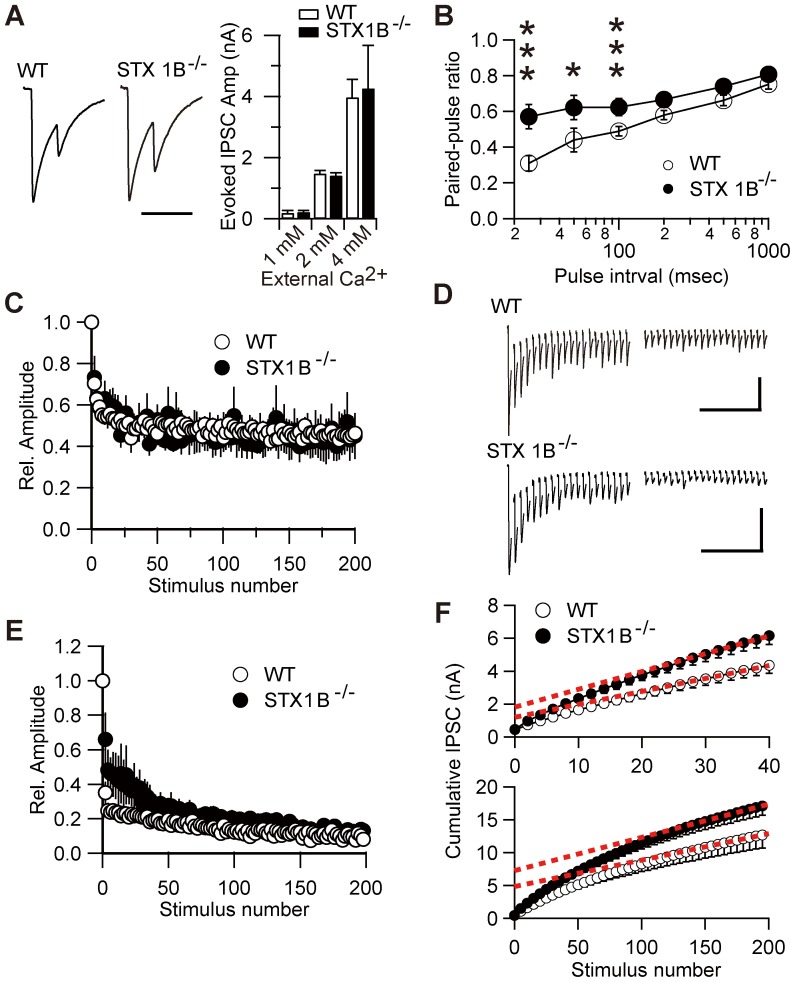
Properties of evoked IPSCs in STX1B null neurons. (A) Representative mean eIPSC waveforms in WT and STX1B^−/−^ synapses (left). Neurons were stimulated at 25 ms interval. Calcium dependence of eIPSCs (right). Average amplitudes of eIPSCs from STX1B^−/−^ (*n* = 6) and WT (*n* = 9) mice were monitored in various concentrations of free extracellular calcium. Scale bar: 50 ms. (B) Paired-pulse ratio of IPSCs was significantly different in cultured neurons from STX1B^−/−^ and WT mice (WT, *n* = 22; STX1B^−/−^, *n* = 20; *p* = 0.008, one-way repeated measure ANOVA with two sample *t*-test). (C) The amplitude of individual eIPSCs during the stimulus train triggered by 200 stimuli at 1 Hz from STX1B^−/−^ (*n* = 6) and WT (*n* = 8) mice, normalized to the amplitude of first response and plotted as a function of stimulus number. (D) Representative eIPSCs train stimulated by 20 Hz recorded from a WT (top) and STX1B^−/−^ (bottom) neuron. The first (left) and last (right) twenty responses are presented. Stimulus artifacts were truncated. Scale bar: 0.5 s, 200 pA. (E) The amplitudes of individual eIPSCs during the stimulus train triggered by 200 stimuli at 20 Hz were significantly different between neurons from STX1B^−/−^ and WT mice (WT, *n* = 6; STX1B^−/−^, *n* = 5; *p* = 0.045, one-way repeated measure ANOVA). (F) Mean cumulative IPSC amplitude value from 40 stimuli, 20 Hz trains in WT and STX1B^−/−^ neurons. Data points between stimuli 30 and 40 (top) and 180 and 200 (bottom) were fitted by linear regression and back-extrapolated to time 0 to estimate the vesicle pool size.

Next, we analyzed properties of evoked inhibitory synaptic responses (eIPSCs). The mean amplitudes of eIPSCs were not significantly different between WT and STX1B^−/−^ mice. We also determined calcium dependence of transmitter release in the presence of 0.1 and 4.0 mM external Ca^2+^, and examined short-term plasticity by measuring the paired-pulse ratio of eIPSCs at different interstimulus intervals. Although the loss of STX1B did not affect calcium dependence of GABA release (Fig. 4A), it increased the paired-pulse ratio of eIPSCs (Fig. 4B). We could not examine calcium dependency of the eEPSCs because of uncontrollable recurrent responses at high external Ca^2+^. To further examine the synaptic properties of STX1B^−/−^ neurons, we measured responses to repetitive stimulation. There was no difference in response between WT and STX1B^−/−^ to a 1 Hz train of 200 stimuli (Fig. 4C), but a 20 Hz stimulatory train produced a rapid, strong depression within the first few stimuli, followed by a near-steady state where further depression occurred very slowly in WT neurons (Fig. 4D and E). At 20 Hz stimulation, the early rapid depression was significantly weaker in STX1B^−/−^ neurons than in WT neurons. We then determined the effective pool size of inhibitory synapses from 40 stimuli. No significant difference was found between genotypes in the estimated pool size (Fig. 4F, top; *p* = 0.091, two sample *t*-test) or vesicle recycling rate (*p* = 0.65, two sample *t*-test), whereas the estimate derived from 200 stimuli showed that the pool size was significantly larger in STX1B^−/−^ neurons (Fig. 4F bottom; *p* = 0.036, two sample *t*-test) with no difference in vesicle recycling rate (*p* = 0.31, two sample *t*-test).

In summary, deletion of STX1B differentially affected glutamatergic and GABAergic synapses. At glutamatergic synapses in STX1B^−/−^ mice, the vesicle recycling rate was greater than in WTs, with no difference in effective pool size. However, at GABAergic synapses, synaptic vesicles in the reluctant component was greater in STX1B^−/−^ than WT neurons, with no difference in vesicle recycling rate.

### Deletion of STX1B decreases RRP size in excitatory and inhibitory synapses

**Figure 5:**
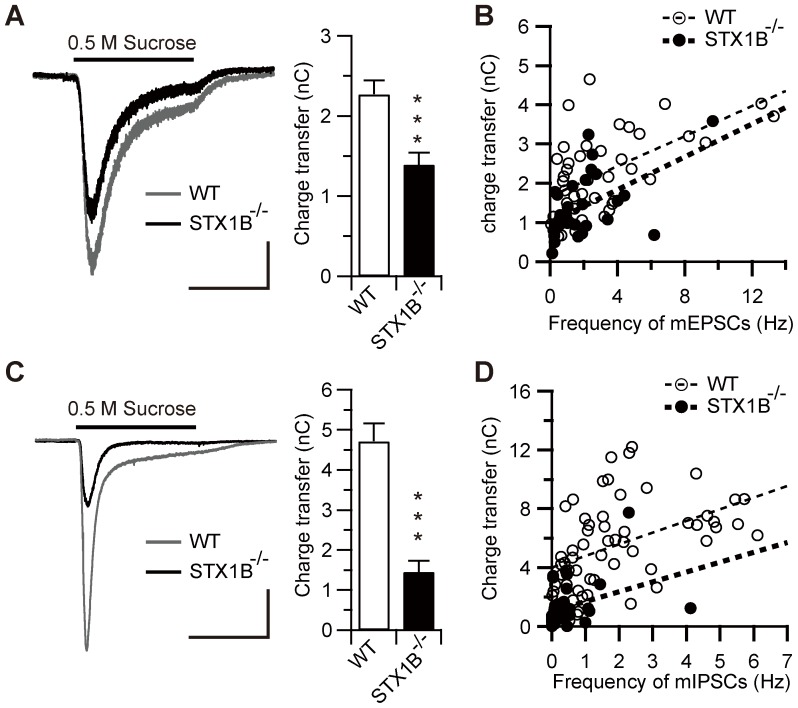
Deletion of STX1B decreases the size of RRP in glutamatergic and GABAergic synapses. (A) Average EPSC evoked by application of 0.5 M hypertonic sucrose solution for 15 s in WT and STX1B^−/−^ neurons (left). Mean RRP size estimated from integral charge transfer induced by application of 0.5 M sucrose solution (right; WT, 2.27±0.17 nC, *n* = 88; STX1B^−/−^, 1.39±0.14 nC, *n* = 62; *p*<0.001, two sample *t*-test). Scale bar: 10 s, 100 pA. (B) Plot of the integral charge transfer as a function of the frequency of mEPSCs. Lines indicate linear fits through data points for each genotype. Although their slopes were not significantly different, the intercept was significantly lower in STX1B^−/−^ neurons (*p* = 0.006, ANCOVA). (C) Average IPSC evoked by application of 0.5 M sucrose solution in WT and STX1B^−/−^ neurons (left). Mean RRP size estimated from integral charge transfer induced by application of 0.5 M sucrose solution (right; WT, 4.72±0.45 nC, *n* = 48; STX1B^−/−^, 1.45±0.28 nC, *n* = 35; *p*<0.001, two sample *t*-test). Scale bar: 10 s, 0.5 nA. (D) Plot of the integral charge transfer as a function of the frequency of mIPSCs. Lines indicate linear fits through data points for each genotype. Although their slopes were not significantly different, the intercept was significantly decreased in STX1B^−/−^ neurons (*p*<0.001, ANCOVA). *** *p*<0.001.

The size of the RRP was also estimated by hypertonic stimulation of the somata and dendrites of recorded neurons. Recently, a number of studies have shown that evoked and spontaneous vesicles form non-overlapping pools with unique molecular signatures [20]–[22]. It is widely believed that application of hypertonic sucrose solution stimulate release of the entire RRP including evoked and spontaneous vesicles in cultured neurons [23], [24], thereby allowing measurement of the total RRP in presynaptic terminals. Hypertonic sucrose solution (+500 mOsm) was applied for 15 s and the integrated charge transfer was used to estimate RRP size. The size of the RRP in glutamatergic (Fig. 5A) and GABAergic synapses (Fig. 5C) of STX1B^−/−^ neurons was, respectively, 61% and 30% that of WTs. These percentage differences were similar to those observed with spontaneous quantal release frequency, where STX1B^−/−^ values were 51% and 27% those of WTs in mEPSCs and mIPSCs, respectively, suggesting that deletion of STX1B selectively decreases the vesicle pool for spontaneous synaptic release. To test this possibility, we studied the relationship between the frequency of spontaneous mPSCs and sucrose-evoked charge transfer. Linear regression analysis revealed a significant correlation between the frequency of mEPSCs and sucrose-evoked glutamatergic charge transfer in WT and STX1B^−/−^ neurons (Fig. 5B; WT, *r* = 0.57, *p*<0.001; STX1B^−/−^, *r* = 0.53, *p*<0.05). Although the slopes of linear regression were not different between WT and STX1B^−/−^ neurons, the intercept was significantly lower in STX1B^−/−^ neurons (WT, 1.67±0.20 nC; STX1B^−/−^, 1.00±0.18 nC, *p*<0.01). Similarly, a significant correlation was found between the frequency of mIPSCs and sucrose-evoked GABAergic charge transfer in WT and STX1B^−/−^ neurons (Fig. 5D; WT, *r* = 0.38, *p*<0.05; STX1B^−/−^, *r* = 0.37, *p*<0.05). The slopes of linear regression were not different between WT and STX1B^−/−^ neurons, but the intercept value was significantly smaller in STX1B^−/−^ neurons (WT, 4.02±0.58 nC; STX1B^−/−^, 1.04±0.29 nC, *p*<0.001). Assuming that the pool size of the evoked vesicles was unchanged in STX1B^−/−^ neurons, these results indicate that deletion of STX1B reduces the number of spontaneous vesicles in the total RRP.

### Synaptic properties of STX1A and 1B double knockout mice

In this paper, we have found that deletion of STX1B changes several presynaptic properties, but that basic synaptic transmission is largely retained. Compared with the deletion of SNAP-25 or VAMP2/synaptobrevin, which causes a substantial reduction in evoked synaptic transmission in mouse central synapses [10], [11], synaptic phenotypes in STX1A or STX1B knockout mice are less severe, possibly because of their mutual complementation.

**Figure 6:**
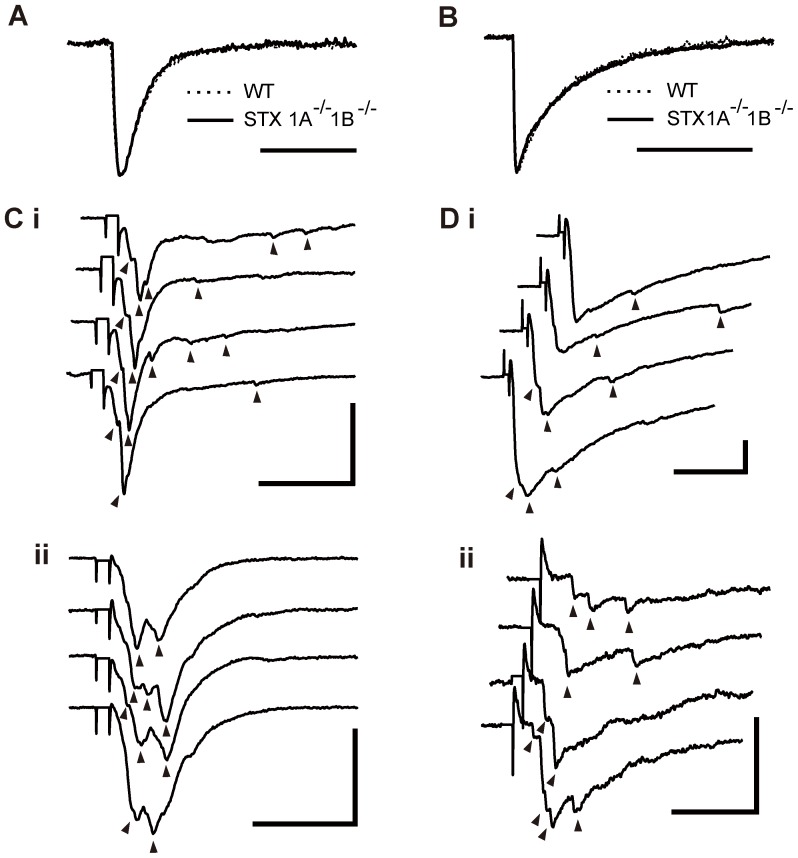
Properties of neurotransmitter release in DKO neurons. (A) Representative mean mEPSC waveforms in WT and DKO synapses. Scale bar: 10 ms. (B) Representative mean mIPSC waveforms in WT and DKO synapses. Scale bar: 40 ms. (Ci-ii) Representative autaptic eEPSC in DKO neurons. Triangles indicate asynchronous component of transmitter release. Stimulus artifacts were truncated. Scale bar: 20 ms, 400 pA. (Di-ii) Representative autaptic eIPSC in DKO neurons. Triangles indicate asynchronous component of transmitter release. Stimulus artifacts were truncated. Scale bar: 20 ms, 200 pA.

To investigate the common neural function of STX1, we produced STX1A/1B double knockout (DKO) mice. Because the DKO mice were embryonic lethal, cortical cultures were prepared from embryonic day (E) 12.5 fetuses. Dissociated neurons derived from a whole cortex of a DKO mouse were plated onto a coverslip. DKO neurons developed normally at first but then degenerated. Only a few neurons survived for 14–21 days in culture. We then performed whole-cell patch clamp recordings, which represented autaptic synapses. We first examined synaptic transmission by measuring spontaneous miniature currents. Three out of 20 neurons tested exhibited mEPSCs. The kinetics of the mEPSCs that can be seen by comparing the peak-scaled average mEPSC waveforms did not differ between WT and DKO neurons (Fig. 6A). Although the mean frequency of mEPSCs (DKO, 3.29±1.19 Hz; *p* = 0.79, two sample *t*-test) was not significantly different between WT and DKO, the mean amplitude of mEPSCs (16.57±2.22 pA, *p* = 0.032, two sample *t*-test) was significantly smaller in DKO neurons. Six out of 20 neurons exhibited mIPSCs. The kinetics of the mIPSCs did not differ between neurons of WT and DKO mice (Fig. 6B). The mean frequency (1.29±0.71 Hz, *p* = 0.039, two sample *t*-test) and amplitude (27.72±7.91 pA, *p* = 0.041, two sample *t*-test) of mIPSCs were significantly lower in DKO neurons. Next, we analyzed evoked synaptic responses on autaptic neurons. Three out of 14 neurons showed autaptic eEPSCs. We found that the amplitude of eEPSCs was significantly smaller in DKO mice than in WTs and, surprisingly, highly asynchronous (Fig. 6C; 396±65.81 pA, *p*<0.001, two sample *t*-test). Ten out of 15 neurons produced eIPSCs which were also attenuated and asynchronous (Fig. 6D; 689.91±29.37 pA, *p*<0.001, two sample *t*-test). Together, our data indicate that STX1 is essential for neuronal survival as well as for synchronized synaptic vesicle exocytosis in glutamatergic and GABAergic synapses.

## Discussion

In the present paper, we report the functional differences between STX1A and STX1B in synaptic neurotransmission by analyzing STX1B^−/−^ mutant mice. A recent study demonstrated functional redundancy between STX1A and STX1B by conducting STX1B knockdown experiments combined with rescue manipulation in neurons from STX1A^−/−^ mice [25]. In this report, severely impaired spontaneous and evoked synaptic transmission caused by STX1 deficiency was rescued both by overexpression of exogenous STX1A or STX1B. However, even though endogenous STX1B protein level was suppressed by RNAi-mediated knockdown, <40% of STX1B protein was still expressed in the cultured neurons [25]. Accordingly, the functional differences between STX1A and STX1B may be unclear by the residual STX1B and overexpressed STX1. In contrast to STX1A knockout mice [12], [13], deletion of STX1B caused death within 14 days after birth. The two syntaxin isoforms are coexpressed in most of the central and peripheral nervous systems [26], [27]. However, nerve terminals of sensory neurons reaching the spinal cord are particularly rich in STX1A, and motor endplates and muscle spindles immunostain for STX1B only [27]. The different distribution patterns of the two isoforms may affect the viability of STX1B^−/−^ mice in vivo.

Although the deletion of STX1A did not show specific effects in glutamatergic or GABAergic synapse transmission [13], the deletion of STX1B showed a lower frequency of spontaneous quantal release without changing its kinetics and amplitude in glutamatergic and GABAergic synapses. We also found a reduced number of docked synaptic vesicles in the presynaptic terminals of STX1B^−/−^ neurons. Together, these observations suggest that STX1B has additional function in vesicle distribution besides regulating spontaneous quantal release. Moreover, the deletion of STX1B caused approximately 70% reduction in the expression level of Munc18-1. It was reported that the binding of Munc18-1 to STX1B is important in sustaining Munc18-1 levels [28], [29]. Although both STX1 isoforms bind to Munc18-1 [30], there is a possibility that STX1A has a lower affinity for Munc18-1 and the deletion of STX1B induces the reduction in Munc18-1 levels and subsequent decrease of spontaneous release rate. Further studies are needed to elucidate the binding properties between each STX1 and Munc18-1.

The frequency of spontaneous synaptic release is affected by release probability and number of synaptic vesicles in presynaptic terminals [31]. We found that the frequency of spontaneous release was related to the RRP size as estimated by sucrose application (Fig. 5B and D). Deletion of STX1B resulted in a smaller RRP, consistent with the relationship between the size of the RRP and frequency of spontaneous release. This indicates that deletion of STX1B reduced RRP size and increased the spontaneous quantal release probability in glutamatergic and GABAergic synapses. Despite the lack of effect on the amplitude of evoked release, significant changes were found in short-term plasticity in glutamatergic and GABAergic synapses of STX1B^−/−^ neurons. The differences were most prominent in shorter pulse intervals. The simple explanation is that deletion of STX1B induces both a decrease in release probability and an increase in vesicle pool size. This assumption agrees with the data from glutamatergic and GABAergic synapses. However, in glutamatergic synapses, we could not estimate the size of the vesicle pool from 40 stimuli at 20 Hz in STX1B^−/−^ neurons. Moreover, estimation obtained from the slope of cumulative release plots indicated that replenishment might be significantly faster during the stimulatory train in STX1B^−/−^ glutamatergic synapses. It is reported that STX1 is implicated in endocytosis [32] and axonal transport [33], [34] of synaptic vesicles, suggests that STX1B plays a role in vesicle replenishment. Another plausible explanation for changes in short-term plasticity is that STX1B deficiency accelerates the replenishment of synaptic vesicles, and then facilitate paired pulse ratio in evoked glutamatergic synaptic transmission without affecting the release probability or the vesicle pool size. Although these explanation seems to contradict the spontaneous release data, recent evidence suggests that spontaneous and evoked release are mechanistically distinct and differentially regulated in central synapses [20]–[22]. The mechanism by which sucrose induces release of the RRP in a Ca^2+^ independent manner remains poorly understood [23]; however, sucrose-evoked vesicle release simply accesses all docked vesicles, while train stimulation may deplete only a subset of vesicles [19]. These stronger neural phenotypes in STX1B^−/−^ compared with STX1A^−/−^ indicate that there are functional differences between STX1 isoforms, and that STX1B is a principal mediator in fast synaptic transmission.

Finally, we investigated the common neural function of STX1A and STX1B in central synapses by analyzing phenotypes of STX1A/1B double knockout neurons. Recently two papers reported STX1 function in synaptic transmission through different animal models for reducing total STX1 levels used in our study [25], [29]. Our DKO mice offer advantages to investigate STX1 function more precisely because even though STX1 protein level was suppressed by RNAi method, other models still have <40% of residual STX1B expression [25] or a low level of STX1B expression in active zone [29]. Consistent with these papers, our results indicated that STX1 deficiency severely impaired synaptic transmission. However, we found via DKO mice the roles of STX1 in Ca^2+^-triggered synchronous release and in neuronal viability. The phenotypes of the DKO neurons were similar to SNAP-25^−/−^ neurons in several apparent aspects such as embryonic lethality, reduced neuron survival in culture and smaller amplitude of spontaneous events [10], [35], [36]. Cleavage of STX1 or SNAP-25 by botulinum neurotoxin induces degeneration of neurons, suggesting that STX1 and SNAP-25 cooperate to support neuron survival [37]. In addition, exogenous expression of other homologous SNARE proteins, syntaxin 2/3/4 and SNAP-23, can substitute STX1/SNAP-25 and prevent toxin-induced neuron death [37], suggesting that syntaxin 2/3/4 might also compensate the functions of STX1 in spontaneous and evoked synaptic transmission in the DKO neurons. STX3 may be a suitable candidate because it was reported that STX3 is required for the exocytosis of synaptic vesicles in ribbon synapses of the retina [38], [39], and for regulated AMPAR exocytosis during LTP in hippocampal neurons [40]. The evoked asynchronous release observed in the DKO neurons is similar to that in synaptotagmin-1 knockout [41], [42] and SNAP-25 knockout neurons expressing exogenous SNAP-23 [36]. These observations suggest that coupling between STX1, SNAP-25 and synaptotagmin-1 is essential for Ca^2+^-triggered synchronous release but not for evoked asynchronous release or spontaneous release. Future studies will be necessary to investigate these possibilities.

In conclusion, our data indicate that STX1 isoforms partly share common functions for neural survival and for synaptic vesicle exocytosis as a t-SNARE. However, importantly, STX1B is primarily involved in the regulation of different types of fast synaptic vesicle exocytosis, including spontaneous and evoked release of glutamatergic and GABAergic synaptic transmission.
